# Vitamin D deficiency among adult patients with tuberculosis: a cross sectional study from a national referral hospital in Uganda

**DOI:** 10.1186/1756-0500-6-293

**Published:** 2013-07-25

**Authors:** Davis Kibirige, Edrisa Mutebi, Richard Ssekitoleko, William Worodria, Harriet Mayanja-Kizza

**Affiliations:** 1 Department of Medicine, Makerere University College of Health Sciences, Kampala, Uganda; 2 Endocrine Unit, Mulago National Referral and Teaching Hospital, Kampala, Uganda; 3 Infectious Diseases Unit, Mulago National Referral and Teaching Hospital, Kampala, Uganda; 4 Pulmonology Unit, Mulago National Referral and Teaching Hospital, Kampala, Uganda; 5 Department of Medicine, Makerere University College of Health Sciences, PO BOX 7062, Kampala, Uganda

**Keywords:** Vitamin D deficiency, Tuberculosis, Uganda

## Abstract

**Background:**

Vitamin D deficiency has been reported among patients with tuberculosis in Africa despite abundant sunshine. Vitamin D plays a fundamental role in improving anti tuberculosis immunity, reducing progression and severity of TB in humans.

**Methods:**

In this descriptive cross sectional study, 260 hospitalized adults with a confirmed diagnosis of TB were enrolled into the study from the pulmonology wards of Mulago national referral and teaching hospital, Uganda. The serum concentrations of 25-hydroxyvitamin D or 25 (OH) D were determined by an electrochemilumniscence immunoassay. Vitamin D deficiency, vitamin D insufficiency, severe and very severe vitamin D deficiency were defined as serum 25(OH) D concentrations of ≤ 20 ng/ml, 21–29 ng/ml, < 10 ng/ml and <5 ng/ml respectively.

**Results:**

Majority of the study participants were males (146, 56.2%) and < 35 years (154, 59.2%). The mean age ± SD was 34.7 ± 9.5 years. Two hundred eight (80%) patients were HIV co-infected with a median CD4 count of 68 cells/mm^3^ (IQR: 17–165). The prevalence of vitamin D deficiency, vitamin D insufficiency, severe and very severe vitamin D deficiency among the hospitalized adult tuberculosis patients was 44.2%, 23.5%, 13.5% and 4.2% respectively. The median (IQR) vitamin D concentration in ng/ml was 22.55 (14.59-33.31).

Vitamin D deficiency was more prevalent in patients with hypoalbuminemia (97.4%), anemia (86.1%), HIV co-infected patients with CD4 count <200cells/mm^3^ (83.2%) and hypocalcemia corrected for serum albumin levels (67%).

**Conclusion:**

Vitamin D deficiency is very common among hospitalized adult tuberculosis patients in Uganda especially in patients with hypoalbuminemia, anemia, HIV co-infected patients with CD4 count <200cells/mm^3^ and hypocalcemia corrected for serum albumin levels.

## Background

Uganda has one of the highest burdens of tuberculosis (TB) in the world with an estimated TB prevalence rate of 193/100,000 in 2010 [1]. It is estimated that 1 billion people worldwide have either vitamin D insufficiency or deficiency [2]. Vitamin D deficiency has been widely documented among African immigrants in Europe [3,4] and African TB patients [5-9]. Currently, there is adequate evidence to suggest that vitamin D deficiency is highly associated with TB [10].

The earliest study assessing the concentrations of serum vitamin D among TB patients in Africa was done by Davies et al. among Kenyan subjects in 1987. Lower concentrations were noted among patients with untreated pulmonary TB compared to the healthy controls [11]. Ensuing studies among African TB patients done in Uganda [5], Guinea Bissau [6], Malawi [7], Tanzania [8] and South Africa [9] have reported similar findings with the documented prevalence of vitamin D deficiency ranging from 8.5% in Guinea Bissau [6] to 62.7% in South Africa [9].

This study aimed to determine the prevalence of vitamin D deficiency and associated clinical factors among adult TB patients admitted on the pulmonology wards of Mulago hospital in Kampala, Uganda.

## Methods

### Study design and patient population

This was a descriptive cross sectional study conducted on the pulmonology wards of Mulago national referral and teaching hospital in Kampala, the capital city of Uganda. Uganda lies in the tropics with constant sunshine all year round and very short rainy seasons. Kampala city is located near the equator at latitude of 00°20´North. The hospital serves a population from predominantly urban and peri-urban areas.

**Figure 1:**
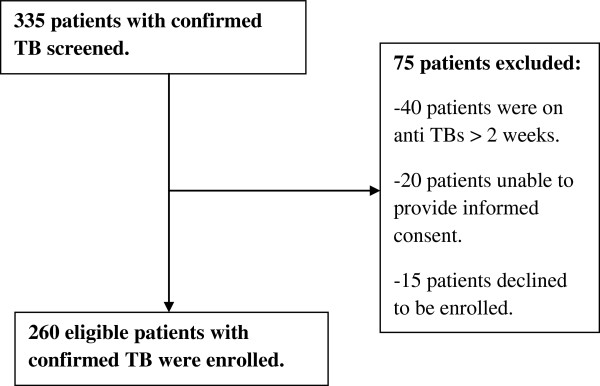
Study screening and enrolment chart.

Eligible consenting adults with a newly confirmed diagnosis of TB were consecutively enrolled during the study period of September 2011 up to February 2012 (Figure 1). A confirmed diagnosis of TB was made in patients who presented with clinical symptoms suggestive of TB and at least one of the following: a positive sputum smear on Ziehl Nielsen or flourochrome (Auramine-O) stain for acid fast bacilli (AFB), a positive Xpert/RIF-TB test result, a positive sputum culture for TB, a positive Ziehl Nielsen stain or flourochrome (Auramine-O) stain for AFB on fine needle lymph node aspirate and a histological diagnosis of TB on lymph node or pleural biopsy. We excluded patients that had been taking anti TB drugs for ≥2 weeks.

These patients were classified as having pulmonary TB (PTB) or extra pulmonary TB (EPTB) and newly diagnosed TB or TB relapse at recruitment. All enrolled patients were screened for HIV.

### Data collection

After obtaining informed consent, pre-tested questionnaires were used to collect information about the socio-demographic data, medical and drug history including use of steroids, multi vitamin supplementation, anticonvulsants and highly active anti retro viral therapy, karnofsky score and the laboratory variables of all the enrolled study participants. All participants also underwent baseline anthropometric measurements of weight and height for calculation of the body mass index (BMI).

Prior to initiation of anti TB treatment, blood was drawn for measurement of the serum 25(OH) D concentrations, complete blood count, CD4 count, serum albumin and calcium concentrations, HIV serology testing, renal function tests (serum urea and creatinine) and liver function tests (alanine transaminase (ALT) and alkaline phosphatase (ALP) concentrations).

### Laboratory analysis

Blood was obtained from the consented study participants and immediately transported to the clinical chemistry and haematology laboratories of Mulago hospital. HIV screening was done using the Determine Rapid HIV-1/2 assay (Abbott Laboratories, Abbott Park, IL, USA), HIV-1/2 STAT-PAK Dipstick assay (Chembio Diagnostic Systems Inc, New York, NY, USA) and Uni-Gold assay. The collected blood samples were centrifuged and the extracted plasma was stored at −20°C. The analysis was done using a full automated COBAS® integra 400 (Roche Diagnostics GmbH) machine.

The serum 25 (OH) D concentrations were determined by an immunoassay technique using the Elecsys vitamin D_3_ assay. It is an electrochemilumniscence immunoassay supplied by Roche diagnostics. It measures the serum 25 (OH) D concentrations in the range of 4–100 ng/ml. Its intra-assay and inter-assay CV was 4.1%-5.7% and 6.6%-9.9% respectively.

The formula below was used in correcting serum calcium concentrations for the albumin levels. Normal serum albumin concentration was defined as a level of 40 g/L.

Corrected calcium concentration = 0.8 (normal albumin − patient’s albumin) + serum calcium in mg/dl [12].

### Statistical analysis

Data was entered into an access data base, FoxPro for windows (Version 2.6; Microsoft) and Stata software, version 12.1 was used for all statistical analysis. Patient characteristics were reported as frequency and percentage for categorical variables, mean and standard deviation (SD) for the normally distributed continuous variables and median and inter-quartile range (IQR) for continuous variables which were not normally distributed.

Based on a sample size of 146 obtained using the prevalence of vitamin D deficiency among pulmonary TB subjects in a Tanzanian study of 10.6% [8] with 80% power and a 2-sided α < 0.05, the statistical power was adequate to detect a medium sized effect of Tuberculosis on Vitamin D deficiency (W of 0.165).

Vitamin D status of the study participants was defined as per the Endocrine Society clinical practice guidelines on evaluation, treatment and prevention of vitamin D deficiency by Holick et al. [13]. Vitamin D deficiency, severe vitamin D deficiency, very severe vitamin D deficiency, vitamin D insufficiency and normal vitamin D levels were defined as serum 25(OH) D concentrations ≤ 20 ng/ml, < 10 ng/ml, <5 ng/ml, 21–29 ng\ml and ≥ 30 ng/ml respectively. Hypoalbuminemia was defined as a total serum albumin level of < 35 g/L while hypocalcaemia as serum calcium concentration corrected for albumin < 9 mg/dl.

### Ethical consideration

The study was approved by the department of Internal Medicine, Makerere College of Health Sciences and Makerere University School of Medicine research and ethics committee. All patients gave informed consent prior to enrolment into the study.

## Results

### Socio-demographic, clinical and laboratory characteristics of the patients

Of the 260 study participants enrolled, majority were male (146, 56.2%) and < 35 years (154, 59.2%). The mean (SD) age of the study participants was 34.7 (9.5) years. Two hundred eight participants (80%) were HIV sero positive and these had a median CD4 of 68(IQR 17–165). One hundred eighty one participants (69.6%) were underweight with a BMI of < 18.5 kg/m^2^. The mean BMI and median haemoglobin level of the study participants was 17.35 (SD 2.51) and 8.9 g/dl (IQR 7.2-10.95) respectively. Hypoalbuminemia and hypocalcaemia corrected for albumin was present in 234 (90%) and 135 (52%) patients respectively. None of the enrolled study participants was on treatment with anti TB drugs, corticosteroids, anti convulsants or any vitamin supplementation (Tables 1 and 2).

**Table 1:** Socio-demographic factors and clinical characteristics of the study participants

**Characteristic**		**Frequency (n = 260)**	**Percentage (%)**
**Age (years) Mean (SD)-34.7 (9.5)**	18-35	154	59.2
	36-50	91	35
	>50	15	5.8
**Gender**	Male	146	56.2
	Gender	114	43.8
**Place of residence**	Rural	77	29.6
	Urban	183	70.4
**Level of education**	No formal education	18	6.9
	Primary level	168	64.6
	Secondary level	54	20.8
	Tertiary level	20	7.7
**Occupation**	Unemployed	58	22.3
	Self employed	34	13.1
	Unskilled	147	56.5
	Skilled	21	8.1
**Smoking status**	Former smokers	66	25.4
	Non smokers	194	74.6
**HIV infection**	Positive	208	80
**History of TB treatment**	No	207	79.6
**Karnofsky score**	<70%	132	50.8
**Category of TB**	PTB	197	75.8
**Body mass index**	<18.5 kg/m^2^	181	69.6
**Mean (SD)-17.4 (2.5)**			
**Dietary intake of fish and milk**	Once weekly	152	58.5

**Table 2:** Laboratory findings of the study participants

**Laboratory characteristic**		**Frequency (n = 260)**	**Percentage (%)**
**Serum calcium corrected for albumin level (mg/dl)**	<9	135	52
**CD4 count (cells/μl)** **Median (IQR)-68 (17–165)**	<200	163	78.4
**§ALT level (U/L) Median (IQR)-11 (6–19)**	≥80	9	3.5
**δ ALP level (U/L) Median (IQR)-113 (79–183.5)**	≥129	109	41.9
Hb** level (g/dl) Median (IQR)-8.9 (7.2-10.95)	<12	212	81.5
**Mean Cell Volume, fl**	<80	131	50.4
**Parathyroid hormone (pg/ml)**	≥65	6	2.3
**Serum albumin (g/L) Mean (SD)-25.3 (7.3)**	<35	234	90

§ALT- Alanine transaminase.

δ ALP- Alkaline phosphatase.

**Hb-Haemoglobin.

### Prevalence of vitamin D deficiency among the patients

Vitamin D deficiency was present among 115 patients (44.2%). Severe and very severe vitamin D deficiency were noted among 35 (13.5%) and 11 (4.2%) participants respectively. Sixty one (23.5%) and 84 (32.3%) patients had vitamin D insufficiency and normal vitamin D levels respectively. The median (IQR) vitamin D concentration in ng/ml was 22.55 (14.59-33.31).

Vitamin D deficiency was noted to be more common among patients with hypocalcaemia corrected for albumin concentration (67%), HIV co-infection (82.6%), CD4 counts < 200 cells/μl (83.2%), anemia (86.1%) and hypoalbuminemia (97.4%).

## Discussion

In this study, we report a prevalence of vitamin D deficiency of 44.2% among 260 hospitalised adult patients with a confirmed diagnosis of TB at Mulago hospital in Uganda.

This demonstrates that vitamin D deficiency is highly prevalent among admitted adult TB patients in Uganda. These findings are congruent with what has been documented in other published African studies among TB patients [5-9].

The high prevalence of vitamin D deficiency noted among our study population could be probably due to the inadequate dietary intake as reflected by the high frequency of hypoalbuminemia (90%) and low BMI (69.6%) among the study participants. In this study, we did not perform a very comprehensive dietary or nutritional assessment. However, majority of the study participants (58.5%) gave a self report of at least once weekly intake of vitamin D fortified milk and fish which are both rich in vitamin D.

Varying prevalence of vitamin D deficiency has been reported among TB patients in Africa. These disparities could be explained by the varying study definitions of vitamin D deficiency used, difference in the techniques used for measurement of vitamin D concentrations, varying location in terms of latitude of the study sites, seasonal variations, varying dietary habits and frequencies of other co-morbidities among the respective study participants.

The highest prevalence of vitamin D deficiency in Africa has been documented by studies done in South Africa (62.7%) [9] and Malawi (42.2%) [7]. The prevalence of vitamin D deficiency reported in our study is comparable to that noted in the Malawian study. Both studies used a similar technique of measuring serum vitamin D concentrations (the electrochemilumniscence immunoassay) and had a comparable prevalence of HIV co-infection among the study participants.

A relatively higher prevalence of 62.7% was documented in a study among a cohort of black patients with active TB done in Cape Town, South Africa [9]. This varying finding with our study could be explained by the differences in sunshine exposure due to the varying latitudes and the seasonal variations in South Africa. Cape Town is located on latitude 33°55´South while Kampala is near the equator with plenty of regular sunshine all year round.

Conversely, studies done in the western part of Uganda [5], Guinea Bissau [6] and Mwanza in Tanzania [8] have reported lower prevalence of vitamin D deficiency among TB patients of 7%, 8.5% and 10.6% respectively compared to ours. A lower prevalence of HIV co-infection (47.2%) in Tanzania, reflecting a better overall health status and the increased consumption of fish among the study participants in Tanzania and Guinea Bissau could explain the lower prevalence of vitamin D deficiency reported from these two settings. A different operational definition for vitamin D deficiency of < 12 ng/ml as per the United States Institute of Medicine guidelines [14] was used in the Ugandan study hence the difference in the prevalence.

Increasing age was found to be independently associated with vitamin D deficiency in our study. This finding has not been noted in other published African studies.

Vitamin D deficiency was noted to be more prevalent in patients with hypoalbuminemia (97.4%), anemia (86.1%), HIV co-infected patients with CD4 count <200cells/mm^3^ (83.2%) and hypocalcemia corrected for serum albumin levels (67%).

In another Ugandan study [5] and a South African study [9], vitamin D deficiency were associated with BMI. Studies from Guinea Bissau [6] and South Africa [9] also noted an independent association between hypovitaminosis D and ethnicity (Fula ethnic group) and seasonal variations respectively.

Our study also demonstrated a very low prevalence of secondary hyperparathyroidism of 2.6% among study participants with vitamin D deficiency (2.3% among all study participants). Secondary hyperparathyroidism is invariably associated with vitamin D deficiency [12]. There is a paucity of information on the correlation between parathyroid hormone and vitamin D concentrations among African TB patients.

The probable explanation of the observed low prevalence of hyperparathyroidism in this study is presence of hypomagnesaemia induced functional hypoparathyroidism commonly seen among chronically ill patients. Hypomagnesaemia results mainly from chronic malnutrition, malabsorption due to enteropathy and chronic diarrhea [15,16]. However, we did not measure magnesium concentrations in this study.

### Study limitations

We used an electrochemilumniscence immunoassay which has been shown to underestimate or overestimate vitamin D concentrations. The liquid chromatography-tandem mass spectrometry method of vitamin D measurement which is regarded the most sensitive and accurate is not available in the country [16].

Although we controlled for some of the confounding factors, there is a possibility that we did not control for all possible confounding factors.

Given that this was a cross sectional study, the temporality between TB and VDD cannot be clearly established.

We were unable to assess the dietary intake of vitamin D and exclude some causes of vitamin D deficiency like nephrotic syndrome, chronic pancreatitis and other causes of malabsorption syndrome among the study participants. However, no study participant had clinical features suggestive of these disorders.

One potential bias in this study was selection bias since it was a hospital based study that recruited only admitted patients that are frequently critically ill.

## Conclusion

Despite all year round presence of abundant sunshine, vitamin D deficiency is highly prevalent among hospitalized adult TB patients in Uganda. Further randomized clinical trials to evaluate the effect of oral vitamin D replacement therapy on clinical outcomes and treatment of TB among Ugandan TB patients could help assess a fundamental role of vitamin D in TB.

## Competing interests

The authors declare that they have no competing interests.

## Authors’ contributions

Concept development: DK, EM, RS, WW, HMK, Data collection: DK, Supervision of the study: EM, RS, WW, HMK, Data interpretation and revision of the first and final drafts: DK, EM, RS, WW, HMK. All authors read and approved the final manuscript.
